# Plasmonic Sensors: A New Frontier in Nanotechnology

**DOI:** 10.3390/bios13030385

**Published:** 2023-03-15

**Authors:** Samir Kumar, Sungkyu Seo

**Affiliations:** Department of Electronics and Information Engineering, Korea University, Sejong 30019, Republic of Korea

Plasmonics is the study of surface plasmons formed by the interaction of incident light with electrons to form a surface-bound electromagnetic wave [1]. Plasmons can configure light to nanoscale volumes, making them attractive for developing new technologies with a wide range of applications [2]. The intense fields created by plasmons can dramatically enhance various light–matter interactions, such as transmission [3], Raman scattering [4], and fluorescence [5], and can also lead to significant localized heating in metallic nanostructures, known as thermoplasmonics [6,7].

Surface plasmons are formed when the oscillation of free electrons on the surface of a metallic nanoparticle resonates with the incident light [8]. The resonance frequency of surface plasmons depends on the size, shape, and composition of the metallic nanoparticle, as well as on the dielectric properties of the surrounding medium [9]. Controlling the plasmon resonance frequency and tailoring the plasmon properties for specific applications is made possible by tuning these parameters. Applications of plasmonics can be found in nanotechnology, biophotonics, sensing, biochemistry, and medicine [10]. However, plasmonics has proven particularly useful in the development of biosensors [11]. The Scopus database contains 37,586 articles related to biosensors that have been published since 2018 (as of February 2023). Twenty-five percent of these studies contain plasmonics-related keywords.

Biosensors are devices that use a variety of physical, chemical, optical, electrochemical, and thermal processes to convert biological interactions into valuable information [12,13]. A biosensor has two components: a transducer and a bioreceptor. Bioreceptors are biomolecules that recognize the target analyte, whereas transducers convert this recognition into measurable signals [14]. Electrochemical and electrical transducers are at the forefront of revolutionizing biosensor technology. However, these systems are limited by their dependence on oxidizing/reducing agents [15]. Optical biosensors based on optical transducers play a prominent role in developing advanced biosensors due to their high detection accuracy and cable-free design [16]. Optical biosensors are capable of multiplex detection and remote sensing. Sensors based on absorption, surface plasmon resonance (SPR), and photoluminescence provide a next-generation optical biosensor platform [17,18,19]. There are several optical biosensing techniques which utilize plasmons, including surface-enhanced Raman spectroscopy (SERS), surface-enhanced fluorescence spectroscopy (SEFS), and surface-enhanced infrared absorption spectroscopy (SEIRAS) [20,21,22].

This *Biosensors* Special Issue focuses on the theory and fabrication of plasmonic nanostructures; patterned surfaces; and devices for SPR, SEFS, and SERS-based biosensors. This Issue contains five research articles, five review articles, and one perspective article covering various topics related to fabrication and recent developments in plasmonic biosensors.

Gaur et al. investigated the interplay between SPR and lossy mode resonance (LMR) in this Special Issue. They compared these two types of sensing techniques in terms of sensitivity, detection accuracy, and figure of merit [23]. The SPR effect is a complicated physical phenomenon that occurs when light strikes a conductive layer of noble metal. This phenomenon occurs at the boundary between a medium with a low refractive index (buffer) and a medium with a high refractive index (sensor glass surface) [24]. In most plasmonic biosensors, the SPR effect detects the binding of biomolecules, such as proteins and DNA, to metallic nanoparticle surfaces. An interaction between a biomolecule and a nanoparticle changes the plasmon resonance frequency, which can be measured with a spectrophotometer. Unlike conventional biosensors, SPR-based biosensors offer several advantages, including label-free detection, high sensitivity, and real-time monitoring. In addition to SPR, LMR can also be used for optical sensing. LMR is a phenomenon that occurs when light is coupled into an optical waveguide and interacts with a lossy resonant mode of that waveguide [25]. The interaction between the light and the resonant mode results in a sharp dip in the transmission or reflection spectrum of the waveguide at the resonant wavelength. This effect is commonly observed in optical fibers, planar waveguides, and other structures supporting guided modes. LMR can be used in various applications, including sensing, filtering, and lasing. By detecting changes in the resonant wavelength, LMR sensors can measure changes in the refractive index, temperature, or other physical parameters in the waveguide environment [26]. The geometric configuration and the material supporting the SPR and LMR are key factors determining its performance. The authors investigated the properties of the bilayer (ITO + Ag structures) and trilayer (ITO + Ag + ITO structures) fiber-optic probes based on ITO, which enables simultaneous excitation of SPR and LMR. They found that LMR showed better sensitivity than SPR in the bilayer configuration. Moreover, as the thickness of the Ag layer increases, SPR depression becomes insensitive, and only LMR depression can be used for detection. Taking advantage of this property, a self-referencing sensor using the SPR depression as a reference point was proposed. In the three-layer configuration, they found that the first LMR depression in the visible region was less sensitive than the SPR depression when the thickness of the outermost ITO was varied. Conversely, when the thickness of Ag was varied, the resonance wavelength of the SPR depression shifted to the shorter wavelength side; however, the resonance wavelength of the LMR depression shifted to the longer wavelength side. The proposed sensor had a high sensitivity of 14 μm/RIU, good detection accuracy, and a Q-factor.

Label-free detection of biomolecules by localized surface plasmon resonance (LSPR) has excellent potential for point-of-care (POC) testing. SPR occurs at the metal–dielectric interface and is sensitive to changes in the refractive index of the dielectric layer. In contrast, LSPR occurs at the surface of metallic nanoparticles and is sensitive to changes in the local environment around the nanoparticle. LSPR results from the confinement of surface plasmons in nanoparticles (with a size comparable to the wavelength of the light used to excite the plasmons) [27]. In developing LSPR-based POC devices, a key challenge is to produce large-scale LSPR substrates that are reproducible and have high throughput. In a study included in this Special Issue, Kim et al. fabricated wafer-scale LSPR substrates using reproducible high-throughput techniques such as nanoimprint lithography, wet etching, and the glancing angle deposition (GLAD) technique [28]. First, they fabricated hard masks of SiO_2_ nanodots on a transparent sapphire wafer using nanoimprint lithography, which was anisotropically etched with a solution of H_2_SO_4_ and H_3_PO_4_, resulting in a patterned sapphire substrate (PSS). Finally, they fabricated an LSPR substrate with Au on the PSS by GLAD and used it to detect biomolecule binding processes without labeling. The GLAD technique allowed for the formation of Au nanostructures on the PSS due to the PSS’s three-dimensional structure (triangular–pyramidal). The Au nanostructures formed by the GLAD technique showed a red-shifted LSPR peak compared to those formed by vertical deposition. This suggests that the LSPR properties can be controlled by the shape of the PSS and deposition conditions, such as the PSS’s angle and rotation.

GLAD is a physical vapor deposition technique used to fabricate complex nanostructures and thin films with controlled porosity and morphology [29,30]. In this technique, the material is deposited obliquely onto a substrate at a low angle of incidence, typically between 70° and 87°, resulting in highly anisotropic structures with directional growth [31]. Nanostructures fabricated by the GLAD technique exhibit high sensitivity, enhanced optical and catalytic properties, periodicity, and controlled morphology, which makes them attractive for sensory applications [32,33]. In another study in this Special Issue, Yadav et al. provided a detailed overview of recent advances in various nanostructures fabricated via GLAD and their applications in the biomedical field [34]. In addition to discussing the various ways to fabricate nanostructures using the GLAD technique, they also considered the assembly configuration, the effects of different growth parameters, and the advantages of GLAD-based nanostructures over conventional nanoparticles and substrates. In addition, the authors highlighted several advantages of GLAD compared to other chemical deposition processes. For example, GLAD does not require precursor materials, which provides safety from toxic precursors and by-products. The chemical composition and thickness in this deposition can be controlled at the atomic level. Higher temperatures are not required, so this technique can produce heat-sensitive substrates.

Of all the metals reported for plasmonic sensors, Ag and Au are considered the most useful plasmonic materials. They show the naturally lowest ohmic losses at optical frequencies due to their strong plasmon resonances in the visible range, their biocompatibility, and their stability [35,36]. In this Special Issue, Gahlaut et al. presented highlights of recent developments in the field of nanostructured Ag substrates for plasmonic sensing, with a focus on SPR and SERS, over the past decade [37]. They mainly focused on the chemical methods of solution phase synthesis and physical methods such as vapor phase deposition, GLAD, and lithographic techniques for synthesizing Ag nanostructures ranging from nanoparticles to nanocubes, nanotriangles, nanorods, and nanowires. In addition, the authors also discuss the latest spectroscopic techniques, focusing on plasmonic enhancement of biosensing methods such as SPR/LSPR, SERS, SEF, and SEIRAS using Ag as plasmonic material.

Metallic nanoparticles, especially Au, have been widely used in plasmonics due to their excellent plasmonic properties. However, the discovery of two-dimensional (2D) nanomaterials, such as MXenes, has revealed new possibilities for plasmonic applications [38,39]. MXenes are a class of 2D transition metal carbides, nitrides, and carbonitrides that have recently shown promise as materials for plasmonics [40]. They have excellent electrical conductivity, mechanical strength, and chemical stability, which makes them attractive for various applications. One of the advantages of MXenes over metallic nanoparticles is their tunability. The plasmonic properties of MXenes can be tuned in the mid-IR to THz regions of the spectrum, and their optical constants (such as the dielectric constant and refractive index) can be tuned by controlling their chemical composition, surface functionalization, number of layers, and morphology [41]. For example, the wavelength of the plasmon resonance of MXenes can be tuned by changing the thickness or the type of functional group on the surface [42]. MXenes have shown great potential in various plasmonic applications, including biosensing, imaging, and photovoltaics. They can also be integrated with other materials, such as polymers or nanoparticles, to create hybrid plasmonic systems with enhanced optical properties. Some researchers have coined the term “smart MXene” to describe MXene-based hybrid materials that exhibit unique properties related to their applications [43,44]. Smart MXene quantum dots (SMQDs), a new and rapidly emerging class of nanomaterials, are tantalizing candidates for SPR biosensor development given their intriguing optical properties, which include light absorption, photoluminescence, and electrochemiluminescence [43]. In their study, contained in this Special Issue, Mousavi et al. discussed recent advances in ultra-sensitive SPR nano biosensors based on SMQDs [45]. They began with an introduction to SPR and SPR biosensors. Then, they explained and discussed the different types of SMQDs and SPR biosensors based on SMQDs. Finally, they presented the useful characteristics of SMQDs for developing SPR biosensors and their biomedical applications, and discussed the current limitations of these biosensors. Overall, MXene plasmonics is a rapidly growing field with promising prospects for various applications, and it is expected to impact future plasmonic sensors’ development significantly.

In this Special Issue, Park et al. discussed innovative advances in biosensors led by DNA nanotechnology [46]. Nanotechnology has made considerable progress in the last decade, allowing us to overcome the limitations of using DNA as the sole form of genetic material and thus develop a new method for constructing biosensors [47]. DNA aptamers can bind to a specific target with a strong affinity. DNA aptamers and enzymes can be produced via the systematic evolution of ligands by exponential enrichment (SELEX), and can bind to specific targets [48]. Aptamers are short, single-stranded DNA or RNA molecules that can bind to specific targets with high affinity and selectivity, similar to antibodies. Various research groups have presented plasmonic biosensing strategies based on aptamers [49]. In aptamer-based plasmonic sensors, the aptamer is immobilized on a metal surface, such as gold or silver. The binding event between the aptamer and the target molecule causes a change in the optical properties of the metal surface, which can be detected by various optical methods. The optical signal can be measured by various methods, e.g., fluorescence, absorbance, and SPR [49]. This review highlighted recent advances in the development of nucleic acids for the purpose of constructing plasmonic biosensors through studies published over the past five years. The authors discussed applying simple aptamers and origami-shaped structures in SPR/LSPR, SERS, and SEF sensing. They discussed several aspects that should be considered to improve the sensing process, including the nonspecific adsorption of unwanted molecules and the fabrication of homogeneous plasmon surfaces. The combination of aptamers and plasmonics has great potential to improve the sensitivity and selectivity of biosensors and enable the development of new diagnostic tools for various applications.

Plasmonics has made significant advances in the field of SERS, a highly effective analytical technique for identifying and detecting molecules [33,50]. SERS significantly amplifies Raman scattering signals when molecules are near plasmonic surfaces [4]. This enhancement is due to the excitation of LSP in metallic nanostructures, which generate strong electromagnetic fields that interact with molecules. SERS is a surface-sensitive technique that can detect analytes even at the level of single molecules [51]. In this Special Issue, Beeram et al. provide an excellent overview of recent trends in SERS-based plasmonic sensors for disease diagnostics, biomolecule detection, and machine learning techniques [52]. The authors review the work of the past decade and use simplified language to address the needs of an interdisciplinary audience. In the first section, the authors discuss the need for plasmonic sensors in biology and the advantages of SERS over existing technologies. Next, the authors discuss the use of SERS-based biosensors for disease diagnosis, focusing on cancer and respiratory disease detection, such as the recent detection of severe acute respiratory syndrome coronavirus 2 (SARS-CoV-2). They then discuss advances in the detection of microorganisms, such as bacteria, with particular emphasis on plasmonic sensors for the detection of biohazardous materials. Finally, the authors discuss machine learning techniques for identifying, classifying, and quantifying biological signals using SERS. They discuss the various machine learning models that have been developed for the trace detection, signal variation, quantification, and identification of SERS signals, including principal component analysis, support vector machines, partial least squares, decision trees, and convolutional neural networks. Considering that SERS is a complex system with many variables, machine learning techniques can help to identify patterns that no experts can identify.

The development of plasmonic biosensors has revolutionized not only the field of biomedical research and diagnostics, but also food safety and environmental research [53]. This Special Issue also contains research on biosensors for food and environmental contaminants such as mercury [54], tetrabromobisphenol A (TBBPA) [55], and thiamethoxam [56]. Kim et al. developed an Au nanoparticle (AuNP)-based immunochromatographic lateral flow assay (ICA) using the light scattering phenomenon of nanoparticles to rapidly detect mercury in rice, with a detection limit of 20 ng g^−1^ and a cut-off value of 500 ng g^−1^ [54]. The proposed ICA strip can be used to qualitatively determine mercury in rice, and the results agree with conventional instrumental methods such as inductively coupled plasma mass spectrometry. Based on aptamer technology, Yue et al. developed a biosensor system to detect thiamethoxam residues [56]. They developed a colorimetric sensor based on AuNPs to detect thiamethoxam in tea leaves. To screen ssDNA aptamers that bind specifically to thiamethoxam, they used graphene oxide SELEX (GO-SELEX) technology. They increased the amount of GO in the screening process to increase the screening pressure. The researchers applied the aptasensor system to the actual detection of samples. They achieved recoveries of 96.94% to 105.86% and RSD values of 0.41% to 3.76%, indicating that the aptasensor can be used for the rapid and sensitive detection of thiamethoxam residues. In another work, Dai et al. developed a hydrophobic Cu–Ag chip for SERS detection of TBBPA [57]. TBBPA is one of the world’s most widely used brominated flame retardants, but its production and use can affect the environment and human health. Recently, there has been much interest in hydrophobic materials due to their wide range of applications in metal corrosion protection, self-cleaning, oil–water separation, and SERS [58,59]. According to Dai et al., the Cu–Ag chip’s hydrophobicity increases the substrate’s affinity for TBBPA, allowing TBBPA to approach the surface of the SERS substrate and subsequently combine with it via Ag–Br interactions. First, the researchers fabricated the hydrophobic copper-coated fabric with an ordered micro-nanostructure. Then, they constructed a hydrophobic Cu–Ag chip by introducing Ag onto the Cu surface through an exchange reaction. Combined with ultrasound-assisted extraction (UAE), they succeeded in quantitatively detecting TBBPA in electronics. After UAE, they extracted and determined the TBBPA content in the electronics with a detection limit of 2.0 mg kg^−1^ (0.01 mg L^−1^ for the TBBPA solution). SERS offers several advantages over conventional methods such as high-performance liquid chromatography (HPLC), including speed, convenience, and sensitivity.

The COVID-19 pandemic, caused by the novel coronavirus SARS-CoV-2, has resulted in a global public health crisis, affecting millions of people worldwide. Rapid, accurate, and sensitive diagnostic methods are critical for optimal patient care. Recently, SERS has emerged as a promising point-of-care testing technique for detecting various analytes, including viruses [60]. Unlike conventional methods such as polymerase chain reaction, which is limited to the analysis of genetic material, SERS offers high specificity and can detect a wide range of analytes. Furthermore, flexible SERS substrates are becoming increasingly important in practical application research due to their robustness and versatility [61]. In this Special Issue, Mousavi et al. evaluated the importance of the flexible SERS substrate for detecting SARS-CoV-2 [62]. They provided an overview of the flexible SERS substrates used to detect different subtypes of the novel SARS-CoV-2 coronavirus and discussed how this method has the potential to become a point-of-care diagnostic tool. In addition to discussing recent advances in SERS-based COVID-19 detection, they discussed the principles of SERS and their amplification mechanisms, the detection of analytes, and the use of multiplex analysis to detect coronaviruses.

In summary, plasmonic biosensors represent a promising technology for the label-free and sensitive detection of biomolecules. The sensitivity, specificity, and label-free detection offered by plasmonic biosensors make them an attractive alternative to conventional detection methods. This Special Issue on “Plasmonic Biosensors”, published in *Biosensors*, provides a comprehensive overview of recent developments and applications in the field of plasmonic biosensors. The research articles present their original work in developing plasmonic biosensors for detecting various biomolecules, including DNA. In addition, this Special Issue highlights the significant advances in the development of plasmonic biosensors and their potential applications in various fields. This Special Issue also highlights the importance of interdisciplinary research and collaboration in developing plasmonic biosensors. Researchers from diverse backgrounds, including physics, chemistry, and biology, have contributed to progress in this field.

In conclusion, this Special Issue on “Plasmonic Biosensors” provides an excellent platform for researchers to present their latest work and exchange ideas regarding the future directions of plasmonic biosensors. The articles in this Issue demonstrate the enormous potential of plasmonic biosensors for various applications, and we can expect to see further advances in this field. As researchers continue to explore the fundamental principles of plasmonics and SERS, we can expect more exciting developments in this field in the coming years.
